# Survival of Extremophilic Yeasts in the Stratospheric Environment during Balloon Flights and in Laboratory Simulations

**DOI:** 10.1128/AEM.01942-18

**Published:** 2018-11-15

**Authors:** André Arashiro Pulschen, Gabriel Guarany de Araujo, Ana Carolina Souza Ramos de Carvalho, Maria Fernanda Cerini, Lucas de Mendonça Fonseca, Douglas Galante, Fabio Rodrigues

**Affiliations:** a Institute of Chemistry, University of São Paulo, São Paulo, Brazil; b Interunities Graduate Program in Biotechnology, University of São Paulo, São Paulo, Brazil; c Graduate Program in Biomolecular Physics, São Carlos Institute of Physics, University of São Paulo, São Paulo, Brazil; d Brazilian Synchrotron Light Laboratory, Brazilian Center for Research in Energy and Materials, Campinas, São Paulo, Brazil; e Airvantis P&D Ltda., São Carlos, São Paulo, Brazil; f Department of Fundamental Chemistry, Institute of Chemistry, University of São Paulo, São Paulo, Brazil; North Carolina State University

**Keywords:** yeasts, Naganishia friedmannii, Cryptococcus, aerobiology, astrobiology, Atacama, extremophiles, stratosphere, UV light

## Abstract

The high-altitude atmosphere is a harsh environment with extremely low temperatures, low pressure, and high UV irradiation. For this reason, it has been proposed as an analogue for Mars, presenting deleterious factors similar to those on the surface of that planet. We evaluated the survival of extremophilic UV-resistant yeasts isolated from a high-elevation area in the Atacama Desert under stratospheric conditions. As biological controls, intrinsically resistant Bacillus subtilis spores were used. Experiments were performed in two independent stratospheric balloon flights and with an environmental simulation chamber. The three following different conditions were evaluated: (i) desiccation, (ii) desiccation plus exposure to stratospheric low pressure and temperature, and (3) desiccation plus exposure to the full stratospheric environment (UV, low pressure, and temperature). Two strains, Naganishia (Cryptococcus) friedmannii 16LV2 and Exophiala sp. strain 15LV1, survived full exposures to the stratosphere in larger numbers than did B. subtilis spores. Holtermanniella watticus (also known as Holtermanniella wattica) 16LV1, however, suffered a substantial loss in viability upon desiccation and did not survive the stratospheric UV exposure. The remarkable resilience of N. friedmannii and Exophiala sp. 15LV1 under the extreme Mars-like conditions of the stratosphere confirms its potential as a eukaryotic model for astrobiology. Additionally, our results with N. friedmannii strengthen the recent hypothesis that yeasts belonging to the Naganishia genus are fit for aerial dispersion, which might account for the observed abundance of this species in high-elevation soils.

**IMPORTANCE** Studies of eukaryotic microorganisms under conditions of astrobiological relevance, as well as the aerial dispersion potential of extremophilic yeasts, are still lacking in the literature compared to works with bacteria. Using stratospheric balloon flights and a simulation chamber, we demonstrate that yeasts isolated from an extreme environment are capable of surviving all stressors found in the stratosphere, including intense UV irradiation, scoring an even higher survival than B. subtilis spores. Notably, the yeast N. friedmannii, which displayed one of the highest tolerances to the stratospheric environment in the experiments, was recently proposed to be adapted to airborne transportation, although such a hypothesis had not yet been tested. Our results strengthen such an assumption and can help explain the observed distribution and ecology of this particular yeast species.

## Abstract (precis)

Studies of eukaryotic microorganisms under conditions of astrobiological relevance, as well as the aerial dispersion potential of extremophilic yeasts, are still lacking in the literature compared to works with bacteria. Using stratospheric balloon flights and a simulation chamber, we demonstrate that yeasts isolated from an extreme environment are capable of surviving all stressors found in the stratosphere, including intense UV irradiation, scoring an even higher survival than B. subtilis spores. Notably, the yeast N. friedmannii, which displayed one of the highest tolerances to the stratospheric environment in the experiments, was recently proposed to be adapted to airborne transportation, although such a hypothesis had not yet been tested. Our results strengthen such an assumption and can help explain the observed distribution and ecology of this particular yeast species.

## INTRODUCTION

Dispersal of microorganisms in the atmosphere contributes to shaping of microbial biodiversity around the world, including extreme habitats, like high-elevation soils and the Antarctic continent (1–3). Atmospheric transportation allows cells to be carried over long distances beyond geographic barriers, playing a key role in the distribution and ecology of microbes (1). Additionally, microorganisms suspended in the air can impact important meteorological and atmospheric processes, such as cloud formation, precipitation, and cloud water chemistry (4, 5). Due to their importance, airborne microbial communities at different environments and altitudes are widely studied using molecular and cultivation-based methods (6–11). To complement these aerobiological investigations on the ecology of airborne microbes, it is also important to study the survival potential of microorganisms under actual atmospheric conditions in order to better understand how living organisms adapt to air transportation and exposure to high altitudes (12, 13).

Microbial cells suspended in the atmosphere many kilometers above the ground must cope with several stress factors, such as solar radiation, low temperatures, and desiccation (14). Microorganisms are not restricted to the troposphere, the lowest layer of the atmosphere where most weather events occur (up to an altitude of 12 km at the tropics, reaching 18 km near the equator). Aerosol particles, including cells, can be carried further above into the stratosphere by natural meteorological phenomena, like thunderstorms (12, 15–18). At this atmospheric layer, more severe environmental conditions prevail (18). Pressures can reach values below 1/100 of that at sea level, temperatures drop to as low as −70°C, and high UV irradiation is present. With a shorter atmospheric path through absorbing gases (particularly ozone), the unfiltered UV radiation is more intense at the stratosphere, mainly at the damaging UV-B range (280 to 320 nm) (19).

Due to the harshness of the stratospheric environment, some authors have proposed to explore it as an analogue of the surface of Mars on Earth (20, 21). This planet may have harbored habitable conditions in its geological past, including superficial liquid water, but it has since lost most of its atmosphere and is now dominated by a cold and dry climate (22, 23). With a thin rarefied atmosphere composed mostly of CO_2_, its present surface is mostly unprotected from the Sun's UV radiation. Mars is considered an important target for astrobiology, and the exploration of its potential to have harbored life through time is the goal of future space missions. In this context, the study of organisms under Mars-like extreme conditions is additionally relevant to planetary protection research (13, 24).

Information regarding the viability of microbial cells during airborne transportation and survival in the combined high-altitude atmospheric stressing factors (or for cells shielded from solar radiation) are topics that can be addressed experimentally, expanding our current knowledge about the adaptation of airborne microbes. Additionally, investigating the capacity of microorganisms to endure these conditions can help the discovery of novel or more efficient cell protection mechanisms, improving our knowledge about life limits under multiple simultaneous stress conditions (25, 26), such as the ones faced by airborne microbes, especially in the stratosphere. Although several works have reported fungi and yeasts collected from air (6, 27, 28) and in high-altitude atmospheric samples (8, 17, 29), there are few studies that have evaluated the potential of these organisms to survive the stressors found in these environments.

In this work, we have investigated the tolerance to atmospheric and stratospheric conditions of cold-adapted UV-resistant yeasts previously isolated from the slopes of an Atacama Desert volcano at 4,000 to 5,000 m above sea level (30). Interestingly, this particular type of high-elevation environment, with freezing temperatures and high UV intensities, has itself been proposed to represent another Mars analogue site on Earth (30–33). The yeast strains chosen were the black-pigmented Exophiala sp. strain 15LV1 and the seemingly non-pigment-producing Naganishia friedmannii 16LV2 (formerly Cryptococcus friedmannii) and Holtermanniella watticus (also known as Holtermanniella wattica) 16LV1. Intrinsically resistant Bacillus subtilis spores were used as biological controls. For the experiments, two stratospheric balloon flights were performed, as well as controlled laboratory tests with a simulation chamber. The results obtained are discussed from astrobiological and aerobiological perspectives.

## RESULTS

**FIG 1:**
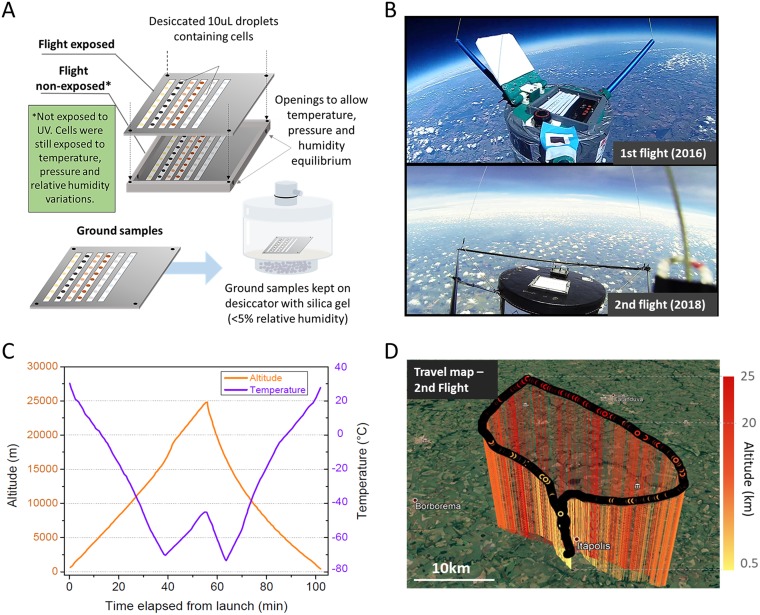
Stratospheric balloon flight experiments. (A) Scheme of the sample holders and all different treatments performed for the microorganisms to be tested. (B) Images of the launches and sample exposure at high altitudes. (C) Altitude and temperature measurements from the second launch (performed in 2018). (D) Travel map of the probe (second launch) during the flight. (Map is from Google Earth, 2018.)

**FIG 2:**
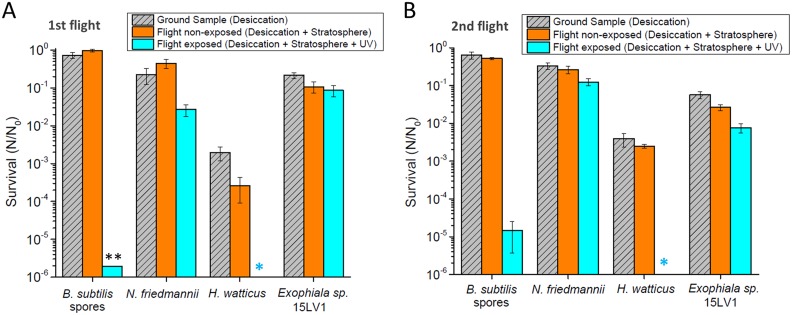
Survival of the tested microorganisms in the stratospheric balloon flights. The tested parameters were desiccation resistance (ground sample), desiccation plus exposure to high-altitude environment but without UV exposure (flight nonexposed), and desiccation plus full exposure to high-altitude environment (flight exposed). (A) Survival of the microorganisms with the first balloon flight, performed in May 2016. (B) Survival of the microorganisms with the second balloon flight, performed in February 2018. Error bars represent the standard deviation between three distinct spots. **, only one spot of B. subtilis spores was recovered from the exposed assay of the first flight; *, no CFU observed for exposed H. watticus on both balloon flights.

The desiccated microorganisms were exposed directly to stratospheric conditions (Fig. 1A and B), and their viability was estimated by CFU enumeration. The two balloon flights lasted for an average of 110 min, reaching up to 25 km altitude (Fig. 1C and D). Additional images of the balloon launches are shown in Fig. S1 in the supplemental material and the microorganisms tested in Fig. S2. The results of the two independent flights are shown in Fig. 2.

As can be observed, the results obtained with the two balloon experiments were similar. Desiccation and exposure to stratospheric conditions without UV nearly did not affect the viability of B. subtilis spores, as expected. However, near 5 log of inactivation occurred in spores exposed to solar UV. The yeasts N. friedmannii and Exophiala sp. 15LV1 were partially affected by the initial desiccation and to the further exposure to stratospheric conditions without UV. However, for the yeast H. watticus, desiccation alone was already enough to reduce viability by 2 log (99% viability drop) (Fig. 2A and B). When considering the full stratospheric exposure (all conditions combined, including UV irradiation), N. friedmannii lost almost 99% viability at the first flight and 90% at the second flight, whereas Exophiala sp. lost 90% and 99% viability in these respective experiments. That is, both of those yeasts suffered a 1- to 2-log viability drop during their passage through the stratosphere. H. watticus was completely inactivated after UV exposure in both flights. Although data are not available for the first balloon flight, we observed at the second flight, in a parallel experiment, that desiccated cells of the yeast Saccharomyces cerevisiae BY4743 also did not survive exposure to the solar UV radiation (Fig. S3).

**FIG 3:**
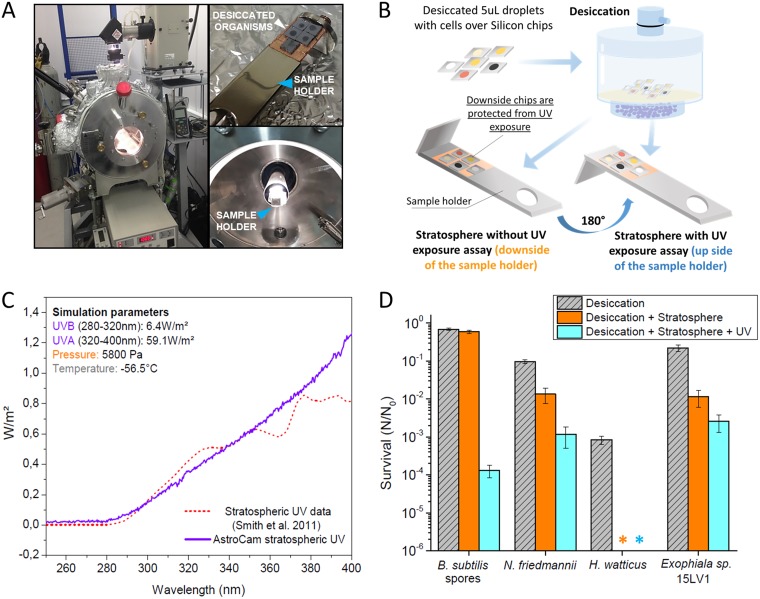
Simulated stratospheric exposure assays. For these assays, cells were exposed for 40 min under a UV flux similar to the one found at 20 km of altitude. (A) Images of the simulation chamber (AstroCam). (B) Scheme of the sample holders and all different treatments performed for the microorganisms to be tested. For the desiccation + stratosphere assay (without UV exposure), an extra protection cover was added over the samples (not shown in the scheme). (C) Simulation parameters and UV spectrum at AstroCam, compared with the spectrum used by Smith et al. (12). (D) Survival of the tested microorganisms under different conditions. Error bars were calculated using triplicates. *, no CFU detected for this treatment.

In addition to the exposures during balloon flights, we also performed simulations of the stratospheric environment using a simulation chamber (AstroCam) capable of reproducing the conditions found at the stratosphere (Fig. 3A). The details of the sample preparation and experimental configuration are shown in Fig. 3B. Due to the characteristics of the simulation chamber (see Materials and Methods for details), cells were desiccated and exposed over silicon chips instead of the polytetrafluoroethylene (PTFE) strips used on the balloon flights. The overall survival behavior observed on the balloon experiments was maintained in the simulation assays, as N. friedmannii and Exophiala sp. 15LV1 survived even after UV exposure, whereas no H. watticus CFU were recovered after irradiation (Fig. 3D). Despite these similarities, data obtained with the simulation chamber differed from those from the two different balloon exposure assays, as the desiccation and desiccation plus stratospheric conditions treatments were more detrimental to yeast cells in our simulation than in the balloon experiments (Fig. 3D). At the simulation chamber, the decompression rates and the cooling speed and conditions are different from the ones at the balloon flights. Therefore, we decided to test whether these parameters were responsible for the observed differences.

**FIG 4:**
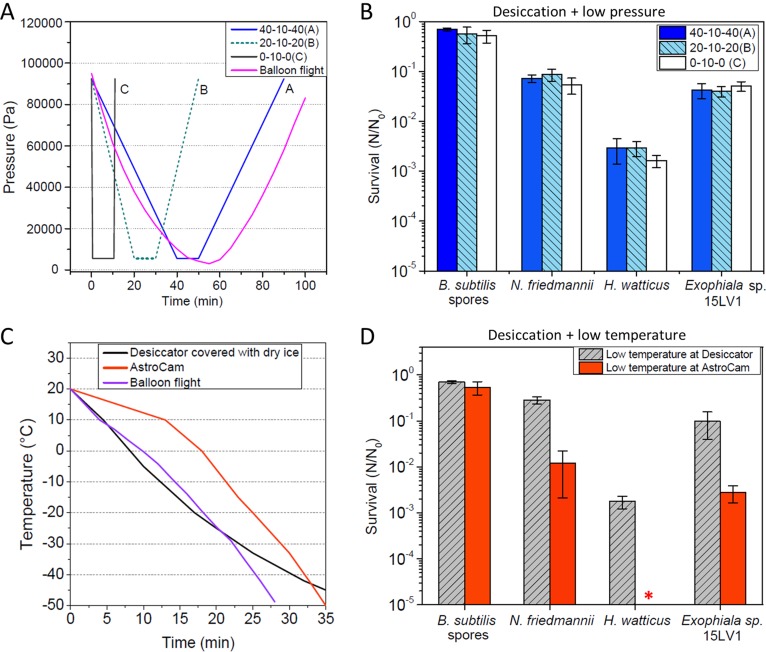
Survival of the tested microorganisms to differential decompression rates and cooling systems. (A) Differential decompression rates used for the assay. Decompression assay A (40 min evacuating, 10 min plateau, 40 min venting) resembles the decompression rates observed at the balloon flight. (B) Survival of B. subtilis spores and tested yeasts to differential decompression rates. No significant differences were observed between treatments. (C) Differential cooling speeds used for the assays, in comparison with the balloon flight. (D) Survival of B. subtilis spores and tested yeasts to cooling down at the AstroCam and at the desiccator covered with dry ice.

Differential decompression rates did not affect the survival of the desiccated yeasts and B. subtilis spores (Fig. 4B). However, when cooling down samples in a desiccator covered with dry ice, higher survival rates (similar to the ones observed during balloon flights) were observed for the yeast cells, whereas a significant reduction in viability was observed at the simulation chamber. These results indicate that the cooling system at the AstroCam is more lethal to yeasts than the low-temperature exposure at the balloon flights.

The viability of Bacillus subtilis spores remained virtually unaffected when exposed to stratospheric parameters while shielded from solar radiation, whereas for all yeasts, even the most resistant ones, a decrease in cell viability during stratospheric conditions without UV irradiation was observed (Fig. 2 to 4). Therefore, when considering a scenario where cells are shielded from UV, spores scored a better survival than with yeasts. However, when considering a scenario where cells are fully exposed to all stratospheric conditions, including solar radiation, N. friedmannii and Exophiala sp. strain 15LV1 scored a greater survival than B. subtilis spores (Fig. 2A and B), even in the simulation chamber assays (Fig. 3D), in which viability was already impaired due to desiccation and exposure to low temperatures (Fig. 4D). H. watticus had a large reduction in its viability, even without solar radiation exposure, and suffered total inactivation when exposed to UV, demonstrating that this yeast does not seem to be adapted to aerial transport and stratospheric exposure, even considering UV-shielded cells.

## DISCUSSION

### Yeast survival in stratospheric environment.

In order to endure atmospheric transportation, microorganisms must deal with intense UV, dehydration, reduced pressures, and low temperatures (12, 18). Desiccation is the first challenge faced by these yeasts, but not for metabolically inert B. subtilis spores, which are extremely resistant to this type of stress (34). In fact, the viability of the spores was observed to be unaffected by desiccation and exposure to additional low pressures and temperatures found in atmospheric high altitudes (Fig. 2 and 3D). The yeasts N. friedmannii and Exophiala sp. 15LV1 were shown to have a reasonable resistance toward desiccation, even as vegetative cells. Survival, as obtained in our results (using a suspension of washed cells), was only achieved by using stationary-phase cultures, high cell densities, and low dehydration rates, factors already known to improve the resistance of yeasts toward desiccation (35). Even the model yeast Saccharomyces cerevisiae, considered to be desiccation tolerant, requires high cell densities at the stationary phase to survive this type of treatment (35, 36). This differs from B. subtilis spores, which can tolerate even abrupt desiccation events.

N. friedmannii 16LV2 and Exophiala sp. 15LV1 achieved a significant survival after direct exposure to the high-altitude atmospheric environment, including UV irradiation, even higher than the survival of B. subtilis spores (Fig. 2A and B). These yeasts were already shown to possess high resistance to UV-C, UV-B, and environmental UV radiation (UV-A + UV-B) under laboratory conditions (30). Considering that UV is the most deleterious factor to cells exposed to high altitudes (12), broad UV resistance probably is a key factor in tolerating the stratospheric environment and might account for the survival observed for our yeasts (Fig. 2A and B). While Exophiala sp. survival may be partially explained by its strong pigmentation, containing both melanin and carotenoids, N. friedmannii 16LV2 is completely pale under our growth conditions, as observed by cell coloration (Fig. S2) and previous Raman spectroscopy analysis (30). It is supposed that other still-uncharacterized protection systems may play a role in this yeast's UV tolerance. The exception of our group of extremophilic yeasts was H. watticus 16LV1, which presented a large loss in viability upon desiccation, increased loss in viability after exposure to low pressure and temperature, and complete loss in viability after UV exposure (Fig. 2). It is important to highlight that our cells were desiccated from suspensions in a 0.9% (wt/vol) NaCl solution, after washing, to eliminate any possible interference and protection of the components of the growth medium in cell survival. In addition, exposures to solar radiation at the stratospheric environment were performed directly, without cover or protection by any component.

Our results using the simulation chamber rendered somewhat different results from the data obtained with the balloon flights (Fig. 3D). The laboratory simulation was somewhat more lethal for the yeasts than the real exposure to the stratosphere (Fig. 2 and 3D), although N. friedmannii and Exophiala sp. 15LV1 still recorded a higher survival than B. subtilis spores at the full simulation with UV exposure. The main observed difference is due to the increased lethality during the desiccation plus stratosphere treatment, which reduced the viability of the tested yeasts 1 to 1.5 log at the simulation chamber (Fig. 3D), compared to 0.2- to 0.3-log reductions at the balloon flights (Fig. 2). For H. watticus, the effects of such treatment were even more severe, in which cells were completely inactivated (Fig. 3D), whereas for the balloon flights, the maximum observed drop was 0.5 log (Fig. 2).

We tested to see if the observed difference may be accounted for by the fact that the decrease in pressure is more abrupt than the one observed at the balloon flight (a couple minutes in the chamber, compared to over 50 min at the balloon flights). However, we measured no difference in survival of desiccated samples exposed to different decompression rates (Fig. 4A and B). Therefore, we aimed to investigate if the cooling system of the AstroCam was responsible for the observed differences. The maximum cooling rates at the chamber are actually lower than the observed rate at the balloon flight (Fig. 4C). Interestingly, we observed that the yeasts' survival inside the cooled desiccator is similar to the observed survival at the balloon flights (Fig. 4D and 2), whereas the cooling system used in the AstroCam was significantly more lethal to the yeast cells (Fig. 4D). Therefore, we believe that the cooling system of the AstroCam accounts for the observed difference between the balloon flights and the simulation, and the cells were actually exposed to a more stressful situation than that at the stratospheric balloon flights.

There are two possible explanations for these changes. The first one is due to the lower cooling rate, which could favor the formation of ice crystals, which are more detrimental to cells. The second explanation is related to a particularity of the AstroCam cooling system, in that only the sample holder (Fig. 3A) is cooled and the remaining parts of the simulation chamber actually remain at room temperature. Due to such characteristics, ice crystals start to form and cover the desiccated cells, even at the low relative humidity inside the chamber (see Materials and Methods). We observed that this ice crystal formation over the samples increases with time and might be responsible for the increased lethality observed at the simulation chamber. Independently of this explanation, an interesting observation can be made, in that B. subtilis spores seem to be indifferent to such variations to low temperature stresses, but yeast cell viability is likely to be more affected.

Importantly, these data highlight that survival in desiccation and low temperature is a key factor dictating the final survival of yeasts to stratosphere exposure, whereas for B. subtilis spores, these factors seem less important (Fig. 4), and UV plays a more relevant role in final survival (Fig. 2). Nevertheless, even considering the more extensive loss in viability to desiccation and low temperature, N. friedmannii and Exophiala sp. 15LV1 still survived exposure to full stratospheric conditions under our simulated conditions, more so than did B. subtilis spores (Fig. 3D), probably due to the remarkably high UV resistance of these yeasts.

### Implications for aerial transportation and astrobiology.

Our results strengthen the recent propositions raised by Schmidt et al. (3, 31) that the species N. friedmannii and members of the Naganishia clade (formerly of the Cryptococcus genus [37]) could be fit enough to survive atmospheric transportation and aerial dispersion, which could explain their abundance in high-elevation soils and its global dispersion. In fact, members of the Naganishia clade have already been found in tropospheric air samples (8) and cloud samples (38), and N. friedmannii is constantly reported in volcanic and mountain soils (3, 32, 33, 39, 40). In our first balloon assay, samples traveled for a distance of over 100 km (Fig. S4), and N. friedmannii displayed good survival even after UV exposure. However, it is likely that cells could survive traveling even farther, especially if partially or totally shielded from UV (if adhered to aerosol particles, for example) or if air transportation occurred at lower altitudes, as for example, at the troposphere. Although no information could be found for airborne cells of the genus Exophiala in the literature, our results suggest that these species could also be properly fit for aerial dispersion.

It is important, however, to note that probably not all extremophilic yeast groups are equally fit for air transportation. As our data showed, H. watticus suffered a great loss in viability already upon desiccation, which is the most critical step for yeasts for airborne transportation (Fig. 2 and 3). Exposure to low pressure and low temperatures decreased cell viability even more (Fig. 4), and finally, UV exposure reduced the survival of H. watticus to essentially zero. It is an interesting observation considering that this yeast, although not as resistant to UV as N. friedmannii 16LV1 and Exophiala sp. 15LV1 under laboratory conditions, was isolated from a high-elevation volcanic area in the Atacama Desert and nevertheless presented a considerable UV resistance in our previous study (30). These results demonstrate the severity and complexity of the stratosphere, which have already been proposed as Mars analogue environments on Earth (20, 21).

Due to the harshness of this environment, microbial survival in the stratosphere is directly correlated with the time that the cells are exposed (13), and hardly any microorganism can endure prolonged unprotected permanence at such high altitudes. Recently, Khodadad et al. (13) exposed Bacillus pumilus spores to full stratospheric conditions at 30 km altitude for a total of 8 h. Even these extremely resistant B. pumilus spores (41) suffered considerable inactivation after 2 h of stratospheric exposure and severe inactivation after prolonged exposure (13). In fact, even when considering the best survival of our yeasts, exposure to the total stratospheric environment still inactivated about 90% of the viable cells of N. friedmannii and Exophiala sp. 15LV1 (Fig. 2, at the second and first flight for each yeast, respectively). Therefore, it is likely that prolonged exposure to stratospheric UV would have a greater impact on the survival of our yeasts, reducing their viability even more. Also, desiccation followed by a low-temperature treatment seems to affect more yeasts than bacterial spores (Fig. 4). Nevertheless, considering that the stratospheric full-exposure assays (Fig. 1 and 2) and the stratosphere simulation (Fig. 3) deeply affected the B. subtilis spores to near-complete inactivation (5-log drop in viability), the survival observed for the yeasts was remarkably high. These results strengthen previous assumptions that yeasts, like N. friedmannii and Exophiala sp. 15LV1, can be good eukaryotic models for astrobiology (30, 31). In fact, an Exophiala species (Exophiala jeanselmei) has already been tested in a Mars simulation, where it presented evidences of metabolic activity under the environmental conditions of Mars (42). In addition, in our previous study, Exophiala sp. 15LV1 scored the highest survival for UV-C and UV-B of our tested yeasts (30), and here, we show that it can endure several conditions found in a Mars-like environment.

N. friedmannii also provides several interesting characteristics for the field of astrobiology, especially when considering Mars' environment. It is expected that on Mars, liquid brines are formed temporarily in regions enriched with salts in contact with water ice (43), which could be permissive for microbial life before water refreezes or evaporates. Also, due to the rarefied atmosphere of Mars, the UV flux on its surface is more harmful than that on Earth, extending from unfiltered UV-B into the shorter wavelengths of the UV-C range (<280 nm) (13). N. friedmannii has shown to be capable of growing during freeze-thaw cycles (40) and can endure considerable UV irradiation at different wavelength ranges, including UV-C (30). N. friedmannii is able to grow at subzero temperatures (−6.5°C) and in a moderate concentration of salts (30). Here, we demonstrate that N. friedmannii can survive even after exposure to all combined stressors found at Mars-like high-altitude environments, as follows: low pressure, desiccation, extremely low temperature, and high solar irradiation. The achieved survival rates were even higher than those of B. subtilis spores, which are commonly studied as potential spacecraft contaminants in the context of planetary protection within the field of astrobiology (24).

Unfortunately, genomic information is still unavailable for this organism. Besides the absence of pigmentation (under our tested conditions) and the already-known ability to perform photorepair (30), no other protection mechanisms of N. friedmannii have been studied to date. Therefore, further investigations should include genome sequencing to enable the identification of possible resistance-related genes. Once sequences are available, deeper molecular investigations, including classic molecular biology approaches (e.g., gene deletion, overexpression, and heterologous gene expression in other yeasts) will be possible, allowing a better understanding of the mechanisms of N. friedmannii to cope with multiple stress conditions and to expand our knowledge about the limits of eukaryotic life.

## MATERIALS AND METHODS

### Strains, media, and growth conditions.

The yeasts Exophiala sp. 15LV1, Naganishia friedmannii 16LV2 (formerly Cryptococcus friedmannii), and Holtermanniella watticus 16LV1, previously isolated from a volcanic region at the Atacama Desert (30), were grown in GYMP broth (glucose, 20 g · liter^−1^; yeast extract, 5 g · liter^−1^; malt extract, 5 g · liter^−1^; NaH_2_PO_4_, 2 g · liter^−1^) at 15°C into the late-stationary phase, which corresponds to roughly 20 days of growth (Fig. S2A). Bacillus subtilis PY79 spores were obtained by growing the cells at 30°C for 4 days in the sporulation medium DSM [Difco nutrient broth, 8 g · liter^−1^; KCl, 1 g · liter^−1^; MgSO_4_ · 7H_2_O, 0.25 g · liter^−1^, completed after autoclaving by adding Ca(NO_3_)_2_ to 10^−3^ M, MnCl_2_ to 10^−4^ M, and FeSO_4_ to 10^−6^ M]. Sporulation efficiency was assayed by phase-contrast microscopy (Fig. S2B) and survival following thermal treatment at 85°C for 15 min. The number of vegetative cells was found to be almost nonexistent (data not shown). Table 1 summarizes the strain characteristics and growth conditions used in this study. CFU were quantified on YM plates (malt extract, 3 g · liter^−1^; yeast extract, 3 g · liter^−1^; peptone, 5 g · liter^−1^; glucose, 10 g · liter^−1^; agar, 20 g · liter^−1^) for the yeasts and on LB plates (tryptone, 10 g · liter^−1^; yeast extract, 5 g · liter^−1^; NaCl, 10 g · liter^−1^; agar, 15 g · liter^−1^) for B. subtilis. The plates were incubated in the dark.

**TABLE 1:** Microorganisms, growth conditions, and approximate number of cells tested for each experiment

Strain	Organism type	Growth medium	No. of cells estimated by CFU counting per spot in:		Notes
			Balloon	Simulation	
Exophiala sp. 15LV1	Yeast	GYMP	4 × 10^5^	2 × 10^5^	UV-resistant yeasts isolated from a high-elevation area on the Atacama Desert (30)
Naganishia friedmannii 16LV2	Yeast	GYMP	4 × 10^5^	2 × 10^5^	UV-resistant yeasts isolated from a high-elevation area on the Atacama Desert (30)
Holtermanniella watticus 16LV1	Yeast	GYMP	6 × 10^5^	3 × 10^5^	UV-resistant yeasts isolated from a high-elevation area on the Atacama Desert (30)
Bacillus subtilis PY79	Bacterium	DSM (sporulation medium)	2 × 10^6^	1 × 10^6^	Spore former

### Sample preparation for the balloon flights.

The cultures were centrifuged, washed twice with a 0.9% (wt/vol) NaCl solution, and diluted, if necessary, to reach a suspension with a final concentration of ∼5 × 10^7^ CFU · ml^−1^ for the yeasts and ∼2 × 10^8^ CFU · ml^−1^ for the B. subtilis spores. Multiple replicates of 10-μl droplets of each of these suspensions were placed over polytetrafluoroethylene (PTFE) strips attached to the balloon's sample holder module with carbon tape (Fig. 1A). The droplets were then slowly dried inside an incubator at 15°C and relative humidity of about 35% before being stored in a plastic container with silica gel to maintain low humidity until launch. The samples were divided between ground samples (desiccation), which were kept inside the container, flight (nonexposed to UV), which were protected from sunlight by a shading cover, and flight (exposed to UV), which were receiving the full solar radiation (direct exposure, without any kind of protection) (Fig. 1A and B). After the flight, the sample holder recovered from the landed payload was placed back inside the container for transportation back to the laboratory. The dried droplet samples were individually resuspended in a 0.9% NaCl solution, diluted, and plated on agar-solidified medium for CFU enumeration. Survival was determined as *N*/*N*_0_, where *N* is the number of CFU recovered after each experiment and *N*_0_ is the initial CFU/concentration of the suspension used for the assays. As an additional control, the washed cells were kept suspended in saline solution for the duration of some of the assays, at 4°C. No loss in viability was observed for these cells (data not shown).

### Balloon flights.

Two independent balloon flights were performed for this work. Instruments housed inside the payload were the Arduino Mega 2560 R3 microcontroller board, a MicroSD 8 GB memory card, a MAX-M8 u-blox global positioning system (GPS) unit, a Ds18b20 temperature sensor, a Bmp180 Gy-68 pressure and temperature sensor, and a Mpu6050 accelerometer and gyroscope. For tracking the probe, two independent Spot Gen 3 systems were used. Images for the launch were acquired using a GoPro Hero3+ Black camera. Helium balloons were used, and after the balloon burst due to high altitude, the probe descent occurred with a parachute system. The first balloon launch (using a Hwoyee 1,200-g balloon) was performed on 14 May 2016, carrying materials for several biological and chemical experiments. The launch was done from the city of São Carlos, São Paulo, Brazil (22°00′18.0″S, 47°56′03.6″W) at 11:30 a.m. local time, with the Sun near its zenith, and landing occurred at roughly 105 km in a straight line from the launching point (21.69′02.88″S, 46.83′54.19″W; Fig. S4). Unfortunately, due to avionics malfunction, environmental data were lost for this flight. It is possible to know, however, that the probe remained above 18 km altitude for approximately 40 min (due to the Spot Gen 3 system, which continued to work), but the maximum altitude reached cannot be ensured; however, we estimate that the probe reached 25 to 30 km altitude, based on similar balloon launches performed later by the group and images acquired (Fig. S1). Also, parts of the exposed samples were lost during landing (spots from B. subtilis and a complete loss of other exposed microorganisms not discussed in this work). The second launch (using a Hwoyee 800-g balloon) was performed on 25 February 2018, from the city of Itápolis, São Paulo, Brazil (21°35′42.59″S, 48°49′59.82″W) at 11:30 a.m. local time. Landing occurred near the launch point (Fig. 1D), which allowed samples to be recovered quickly. Environmental data are shown in Fig. 1C and S5.

### Stratosphere simulation using AstroCam.

Stratospheric environment simulation experiments were performed using a planetary simulation chamber built by our research group (AstroCam, Fig. 3A) capable of recreating most aspects of the high-altitude flight, including low pressure, low temperature, and high UV flux. Simulation parameters were adjusted to represent the conditions at 20 km above sea level. UV fluxes were calibrated according to the UV parameters used by Smith et al. (12), who simulated the 20-km-altitude environment to test B. subtilis survival under stratospheric conditions. UV intensities were measured using a Vilber Lourmart RMX-3W radiometer and an Ocean Optics QE65000 UV-Vis fiber-optic coupled spectrometer. Temperature (−56.5°C) and pressure (5,800 Pa) values were used according to the U.S. Standard Atmosphere model.

Microbial growth and sample preparation were exactly as performed for the balloon flight experiments (described above). However, silicon chip supports were used instead of PTFE strips (Fig. 3B), since the temperature inside the chamber is controlled at the sample holder and silicon is a more efficient temperature conductor than PTFE. The volume of droplets was also reduced from 10 μl to 5 μl. The silicon chips were attached to the sample holder by vacuum-compatible adhesive copper tape and positioned inside the simulation chamber (Fig. 3A). The pressure inside the chamber was lowered by an Adixen ACP15 dry mechanical pump to 5,800 Pa, maintained in-flow through a needle valve with ambient air passed through humidity-absorbing silica gel columns. An ARS CS204PB-450 liquid helium-refrigerated cryostat in contact with the sample holder was used to control the temperature to −56.5°C. The samples were divided between the desiccation controls, which were not taken to the chamber and kept inside a container with silica gel at low relative humidity; desiccation + stratosphere samples, which were exposed to the simulated environment on the underside of the sample holder but protected from the UV radiation by a cover; and desiccation + stratosphere + UV, which received the full simulated solar radiation (direct UV exposure, without any kind of protection) for a total of 40 min. The irradiation was performed with an Oriel solar simulator with a 1,000-W xenon arc lamp equipped with a water filter to attenuate the longer wavelength infrared and an AM1 filter to shape the lamp's emission closer to the solar spectrum with a 1-atm attenuation path. After the simulation, the samples were brought to room temperature, and the chamber was vented with dry air. The samples were processed as described before for the balloon flight experiments. Simulations were performed in triplicate.

### Differential decompression rate assay and differential cooling and freezing assay.

Survival was evaluated for differential decompression rates using a programmable Büchi V-700 vacuum pump with V-855 controller, coupled to a desiccator containing silica gel. Cells were prepared in the same manner as for the simulation chamber assay, where 5-μl droplets were pipetted over silicon chips and slowly desiccated. The samples were then positioned inside the desiccator, which was evacuated and, after a 10-min period, vented at controlled speeds programmed in the pump. Viability was estimated by CFU counting. For a comparison of survival at different cooling rates, cells were grown and washed as described above. Droplets of the same cultures (5-μl droplets) were pipetted over silicon chips and slowly desiccated. A group of silicon chips containing the cells was cooled at the AstroCam simulation chamber at low pressure (maintained in-flow with dry air; see above), whereas another group was placed inside a desiccator, which was then then cooled down by covering it with dry ice inside an insulated box. Both cooling treatments were performed for 2 h. Viability was estimated by CFU counting and plotted as *N*/*N*_0_.

## Supplementary Material

Supplemental file 1
