# Supplementary material for: Survival of Extremophilic Yeasts in the Stratospheric Environment during Balloon Flights and in Laboratory Simulations
Source: Appl Environ Microbiol. 2018 Nov 15;84(23):e01942-18. doi: 10.1128/AEM.01942-18 (PMC6238051; doi:10.1128/AEM.01942-18)
Supplement: Supplemental file 1 [file zam023188865s1.pdf]

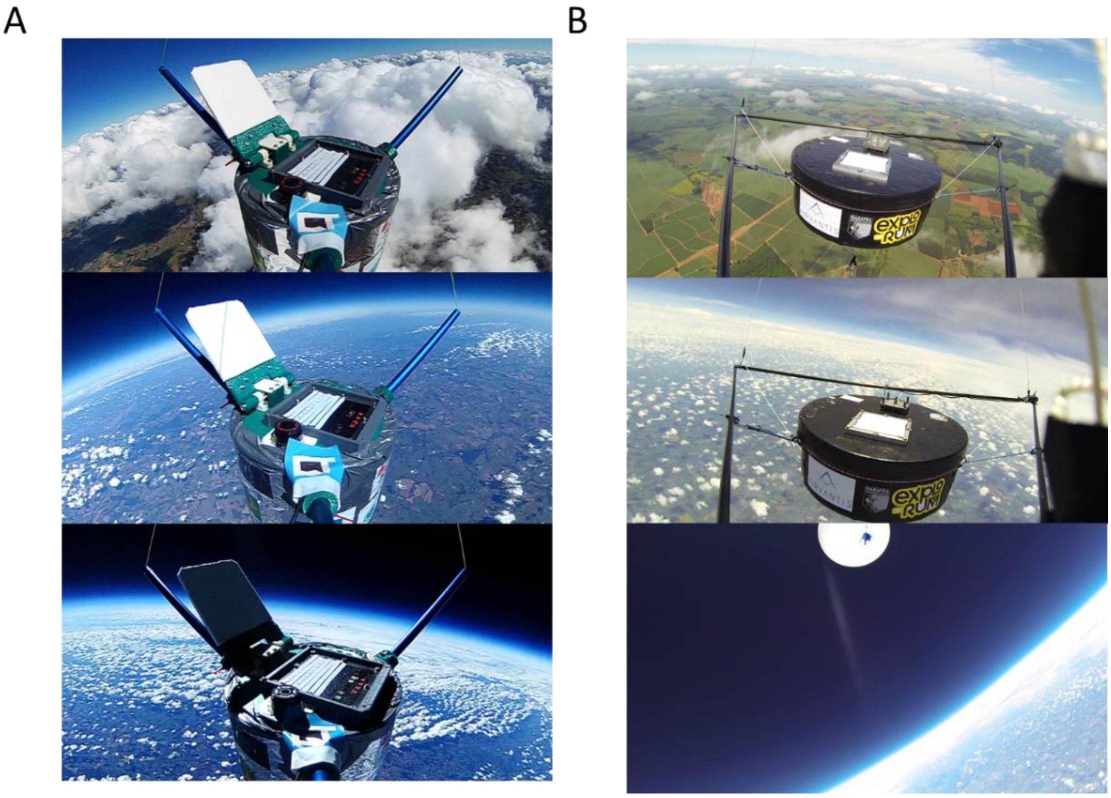

Figure S1. Images of the balloon launch experiments. (A) First balloon flight assay, performed on May 2016. (B) Second balloon flight assay, performed on February 2018. The last image of this launch shows the stratospheric balloon moments before it burst. During the flight, a loosen rope hit the camera, changing its position, and unfortunately no images of the probe at the highest altitude are available.

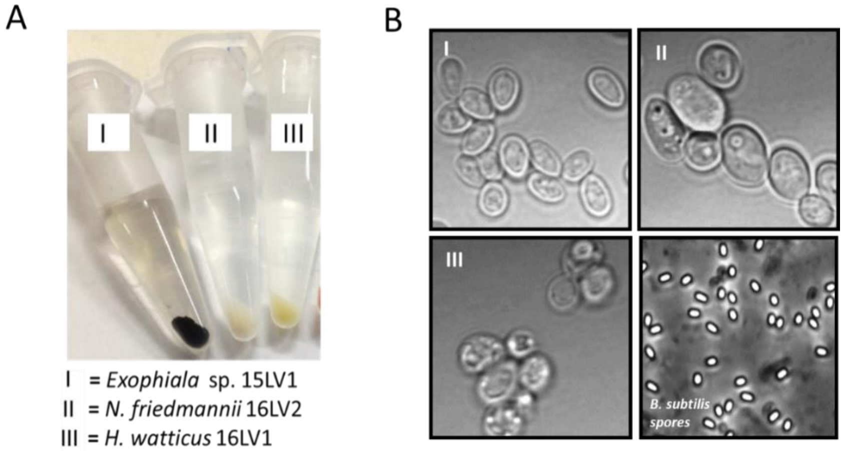

Figure S2. Microorganisms used in the study. (A) Coloration of the cell pellets of the tested yeasts, after the washing procedure. As can be observed, *Exophiala* sp. presents strong black coloration, due to melanin production. However, *N. friedmannii* cells are creamy-white. (B) Microscope observations of the tested organisms. For *B. subtilis* spores imaging, a phase-contrast objective was used. Images were acquired using a Nikon Eclipse TiE Microscope.

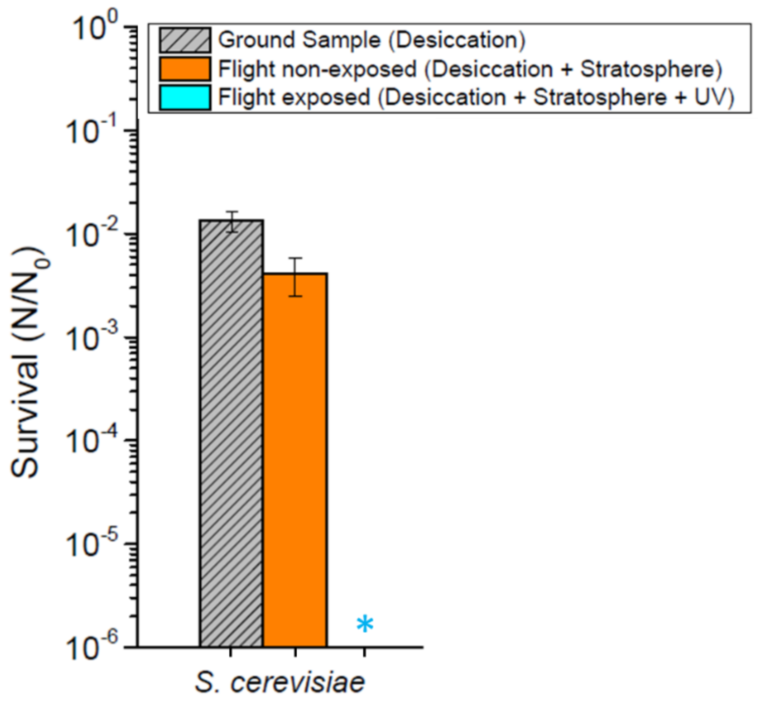

Figure S3. Survival of *S. cerevisiae* BY4743 to the second balloon flight. *S. cerevisiae* was grow on YM liquid media, washed and prepared for the balloon flight the same as the other yeasts.
*= No CFU was detected for this treatment

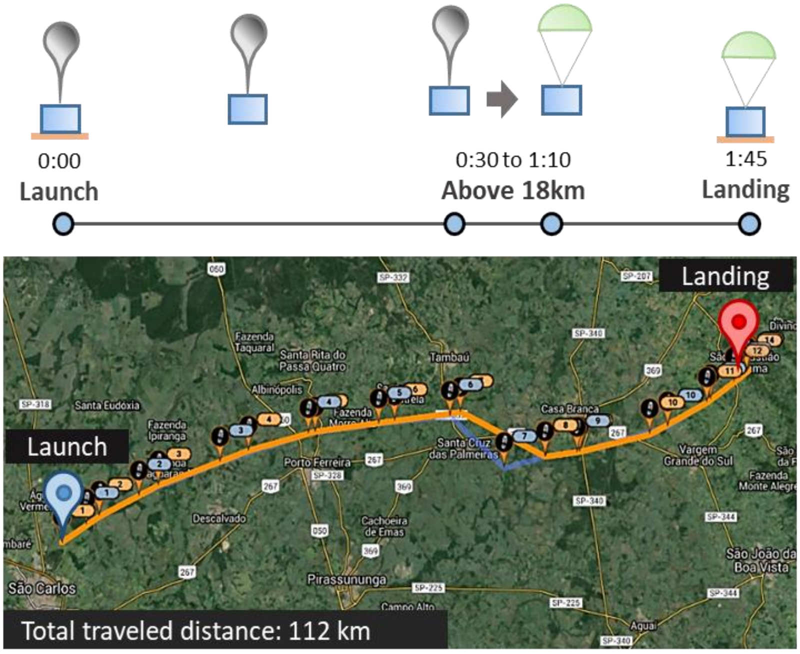

Figure S4. Flight map and scheme of the first balloon flight. Data of the travel were acquired with a Spot Gen 3 system. Map credit: Google Earth, 2016.

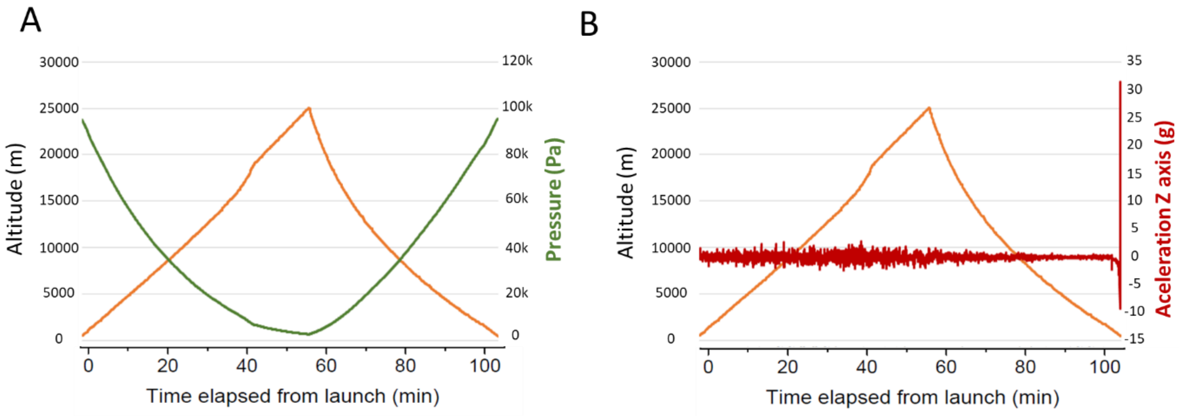

Figure S5. Additional environmental data acquired from the second balloon flight. (A) Plot of altitude and pressure measurements, in function of time (B) Plot of altitude and acceleration, in function of time. The moment in which the probe hits the ground can be seen as a large increase in acceleration.
